# Intracellular *Porphyromonas gingivalis* Promotes the Proliferation of Colorectal Cancer Cells *via* the MAPK/ERK Signaling Pathway

**DOI:** 10.3389/fcimb.2020.584798

**Published:** 2020-12-23

**Authors:** Wenxin Mu, Yiqun Jia, Xiaobing Chen, Haoyu Li, Zhi Wang, Bin Cheng

**Affiliations:** ^1^Hospital of Stomatology, Guanghua School of Stomatology, Guangdong Provincial Key Laboratory of Stomatology, Sun Yat-Sen University, Guangzhou, China; ^2^Stomatology Center, Shenzhen People’s Hospital, The Second Clinical Medical College of Jinan University, The First Affiliated Hospital of Southern University of Science and Technology, Shenzhen, China

**Keywords:** gingipain cysteine endopeptidases, cell cycle, cell proliferation, colorectal neoplasms, *Porphyromonas gingivalis*

## Abstract

*Porphyromonas gingivalis* (*P. gingivalis*) is a keystone pathogen in periodontitis. However, several clinical studies have revealed an enrichment of *P. gingivalis* in the stool samples and colorectal mucosa of colorectal cancer patients. Thus, the goal of this study was to determine whether *P. gingivalis* can promote colorectal cancer progression *in vitro*. We established an acute infection model (24 h, multiplicity of infection =100) of *P. gingivalis* invasion of colorectal cancer cells to study the alterations induced by *P. gingivalis* in the proliferation and cell cycle of colorectal cancer cells. We observed that *P. gingivalis* can adhere and invade host cells a few hours after infection. Once invaded, *P. gingivalis* significantly promoted colorectal cancer cell proliferation, and the percentage of S phase cells was increased in the cell cycle assay. However, KDP136, a gingipain-deficient mutant of *P. gingivalis* 33277, showed a decreased ability to promote colorectal cancer cell proliferation, indicating that gingipain is associated with colorectal cancer cell proliferation. Furthermore, we extracted RNA from colorectal cancer cells for high-throughput sequencing analysis and reconfirmed the results by quantitative polymerase chain reaction and western blot analyses. The results suggested that the MAPK/ERK signaling pathway is significantly activated by *P. gingivalis*, while these changes were not observed for KDP136. In conclusion, *P. gingivalis* can invade cells and promote the proliferation of colorectal cancer cells by activating the MAPK/ERK signaling pathway. Gingipain is an essential virulence factor in this interaction.

## Introduction

The oral microbiota is one of the most complex human microbiomes, second only to that of the gastrointestinal tract, containing 26% of the bacterial species associated with the human body (Group et al., 2009). Furthermore, the results of a recent clinical trial demonstrated that the vast majority of oral microbial species can be transmitted from the oral cavity to the large intestine (Schmidt et al., 2019). Oral bacteria are closely associated with many oral diseases and systemic diseases outside the oral cavity. As the most common opportunistic pathogen in periodontal diseases, Fusobacterium nucleatum (F. nucleatum) is associated with oral squamous cell carcinoma (OSCC) (Al-Hebshi et al., 2017), pregnancy complications (Han et al., 2010), and colorectal cancer (CRC) (Rubinstein et al., 2013). Interestingly, a mixed infection of F. nucleatum and *Porphyromonas gingivalis* (*P. gingivalis*) has been shown to be much more effective than mono-infection in experimental periodontitis (Polak et al., 2009). In addition, *P. gingivalis*, a major pathogen of periodontitis, is also associated with OSCC (Geng et al., 2017; Lafuente Ibanez de Mendoza et al., 2020; Wen et al., 2020), esophageal squamous cell carcinoma (Gao et al., 2016), pancreatic cancer (Michaud et al., 2013), cardiovascular disease (Gibson et al., 2004) and rheumatoid arthritis (Wegner et al., 2010). In the mucosa-adherent and fecal microbiota, *Porphyromonas* has been shown to be enriched in CRC patients (Chen et al., 2012; Ahn et al., 2013; Wu et al., 2013; Zackular et al., 2014).

The virulence factors of *P. gingivalis* include fimbriae, hemagglutinin, capsule, lipopolysaccharide and gingipain. Specially, gingipain plays an essential role in the pathogenicity of the organism in periodontal disease. As a family of unique cysteine endopeptidases, gingipain are abundantly expressed and located on the outer membranes of *P. gingivalis* or secreted into the extracellular milieu (Pike et al., 1994). The gingipain family consists of two types of arginine-specific protease (Rgp; encoded by *rgpA* and *rgpB*) and a lysine-specific protease (Kgp; encoded by *kgp*). Among them, RgpB has been the focus of structural studies aimed at elucidating post-translational processing and maturation of these enzymes because of its simple structure (Eichinger et al., 1999; Nguyen et al., 2007). Gingipain can provide a general proteolytic tool for the degradation of proteinaceous nutrients to *P. gingivalis* for growth. Besides, gingipain had also been proven to be essential in the processing of fimbrial proteins to facilitate bacterial adhesion to the host tissues (Njoroge et al., 1997; Weinberg et al., 1997). Gingipain can also enable bacterial evasion of the host immune response by surface receptor cleavage and cytokine degradation (Brien-Simpson et al., 2003). Previous studies showed that gingipain can activate the ERK1/2-Ets1, p38/HSP27, and PAR2/NFκB pathways to promote cellular invasion and metastasis in OSCC cells (Inaba et al., 2014).

Consequently, we hypothesized that *P. gingivalis* is probably associated with CRC progression and that gingipain is a keystone virulence factor in this process. To test this hypothesis, in this study, we used an acute *in vitro* model of *P. gingivalis* infection of CRC cells.

## Materials and Methods

### Bacteria and Cell Culture

The bacterial strains, P. gingivalis ATCC 33277, P. gingivalis W83 and F. nucleatum 25586 were purchased from ATCC. P. gingivalis KDP136 (ΔrgpAΔrgpBΔkgp), a gingipain-deficient mutant of *P. gingivalis* 33277, were kindly provided by Dr. Jinlong Gao from Faculty of Medicine and Health, the University of Sydney. P. gingivalis were grown in BHI broth supplemented with yeast extract (5 mg/ml), cysteine (1 mg/ml), vitamin K1 (0.5 μg/ml) and hemin (5 μg/ml) in the anaerobic chamber (oxygen concentration < 1%). Human CRC cell line S1 (a clone of LS174T cells) and murine colon cancer MC38 cells were purchased from ATCC. The cells were cultured in DMEM medium (Thermo Fisher Scientific Inc., MA, USA) supplemented with 10% fetal bovine serum (FBS) at 37°C in 5%CO_2._

### Cell Adhesion Assay

To detect the adhesive ability of P. gingivalis, immunofluorescence microscopy and flow cytometry were used. Cells were infected with P. gingivalis at a MOI of 100 for 6 h incubation. Then infected cells were washed with PBS three times and fixed with 4% paraformaldehyde for 30 min at room temperature. The primary antibody against RgpB (a kind gift from Jinlong Gao, 1:200) were added to cells at 4*℃* overnight. Cells were incubated with Alexa Fluor 488 Goat anti-Mouse IgG(H+L) (EMAR, Beijing, China, 1:100) for 1 h at room temperature and photographed by fluorescence microscope (Zeiss Axio observer Z1). Nuclei were stained with DAPI (Solarbio, Beijing, China, 1:100) for 5 min.

P. gingivalis were incubated with FITC (0.1mg/ml) for 30 min at room temperature, followed by washing with PBS three times. Cells were infected with the P. gingivalis labeled with FITC (MOI=100) at 4°C for 30 min, then washed with PBS three times to remove the P. gingivalis in supernatant. The cells were harvested by trypsinization and processed by flow cytometry (Beckman Coulter Cytoflex).

### Cell Invasion Assay

S1 and MC38 cells (6×10^5^) were seeded in 6-well plates and infected with P. gingivalis (MOI=100) for 24 h. Cells were washed three times with PBS and harvested by trypsinization, then fixed with 2.5% glutaraldehyde in 0.1 M PBS (PH=7.4) at 4 °C overnight. Thin sections were cut and stained with uranyl acetate-lead citrate. Followed by observing in transmission electron microscope (TEM, H7650 Hitachi, Japan). Three fields (1000 ×, 30–50 CRC cells per fields) were selected randomly and the number of CRC cells with P. gingivalis invasion and without P. gingivalis were counted separately.

The invasion ability of P. gingivalis toward CRC cells were also measured by an antibiotic protection assay (Lamont et al., 1995; Yilmaz et al., 2002). Cells were plated in 6-well plates at a density of 3×10^5^ cells/well in complete medium for 12 h, following incubating with P. gingivalis (MOI=100) for 24 h. Remaining external bacteria were killed with gentamicin sulfate (Solarbio, Beijing, China, 1 mg/ml) for 90 min. Cells were washed with PBS three times and lysed with sterile distilled water for 30 min. Internal bacteria were released and plated on blood agar supplemented with hemin and menadione and cultured anaerobically. Colony forming units (CFU) of invasive P. gingivalis were then enumerated and invasion efficiency (Invasion efficiency (%) = CFU of *P. gingivalis* inside CRC cells/CFU of *P. gingivalis* in initial inoculum) were expressed to assess the invasive ability of P. gingivalis (Arjunan et al., 2020).

### Cell Counting Kit-8 Assay

Cell proliferation was determined using cell counting kit-8 assay (Telenbiotech, Guangzhou, China). S1 and MC38 cells (3000 cells per well, 100μL culture medium) were seeded in 96-well plates. After 8 h incubation, the cells were infected with P. gingivalis at a MOI of 100 for 24 h. The growth medium in each well was removed, and then filled with cck-8 solutions. After incubated for 1 h at 37*℃*, the optical density value was detected at 450nm with a versatile microplate absorbance reader (Tecan sunrise, Untersbergstrasse, Austria).

We detect the cell viability of colorectal cancer cells pretreated with *P. gingivalis* 33277 (MOI=100) for 0-96 h. the results showed that *P. gingivalis* can promote the proliferation of CRC cells in a time dependent manner in 0–24 h, and the cell viability of CRC cells reached a plateau in 24–96 h incubation. So the acute infection model in this study was designed as 24 h cocultivation (**Supplementary Figure 1**).

The exogenous gingipains were kindly provided by Prof. Min Liang (Guanghua School of Stomatology, Sun Yat-Sen University), they exogenous it from P. gingivalis W83 strain as described in previous report (Sheets et al., 2005; Zhang et al., 2017; Mo et al., 2020).

### Colony Formation Assay

S1 and MC38 were infected with P. gingivalis (MOI=100) for 24 h. Cells were then seeded in 6-well plates (500 cells per well) and cultured for 14 days. Cells were fixed with 4% paraformaldehyde for 30 min at room temperature and stained for 5 min with 0.1%(w/v) crystal violet for 20 min. The number of eukaryotic cell colonies were counted using image processing software (ImageJ 1.48v).

### Cell Cycle Analysis

Cells were harvested and fixed with 70% ice-cold ethanol at 4°C for 16–18 h. Cells were washed with cold PBS then re-suspended with mixed medium (PI: 50ug/ml, RNase: 100ug/ml) for incubation at 37°C; for 30 min. The percentage of the cells in each cell cycle phases were detected by a flow cytometer (ModFit LT 4.0).

### RNA Extraction and Quantitative Real-time PCR

Cells were infected with *P. gingivalis* at a MOI of 100. Cells were washed by PBS and harvested at 3, 6, 12 and 24 h. Total RNAs were extracted using Trizol reagent (Thermo Fisher Scientific Inc, MA, USA). The concentration and quality were measured using a Nanodrop (Thermo Fisher Scientific Inc, MA, USA). The RNAs were then converted into cDNA using primerscript RT-polymerase (Takara, Shanghai, China). The quantification of selected genes was measured using LightCycler96 (Roche, Shanghai, China) and the data was analyzed using the 2^-ΔΔCt^ method. The primers of KRAS, BRAF, MEK2, ERK2, C-Fos, AP1, and GAPDH (Tsingke, Guangzhou, China) are all listed below in **Supplementary Table 1**. GAPDH was used as an endogenous control and the mRNA levels were normalized to GAPDH.

### Western Blot and Antibodies

The total protein was extracted using RIPA Lysis Buffer (Beyotime Biotechnology, Shanghai, China) containing protease and phosphatase inhibitor cocktail (CWBIO, Beijing, China). BCA Protein Assay Kit (BIOTEKE, Beijing, China) was used to quantify the protein. Extracted protein were separated with 8~12% SDS-polyacrylamide gel electrophoresis and transferred to 0.2 μm PVDF Membrane (Roche, Shanghai, China). The membrane then blocked with 5%BSA and incubated with primary antibodies against p-MEK1/2 (CST), t-MEK1/2 (CST), p-ERK1/2 (CST), t-ERK1/2 (CST), p-Akt (308) (CST), p-Akt (473) (CST), Akt (CST), and GAPDH (CST) at 4*℃* overnight. Following washing with TBST three times and exposing to secondary antibodies (CST) for 1 h at room temperature. The blots were visualized using an imaging system (BIORAD ChemiDoc Touch) and quantified by an image processing software (ImageJ 1.48v). GAPDH was used as an endogenous control and the protein levels were normalized to GAPDH.

### Statistical Analysis

**Figure 1:**
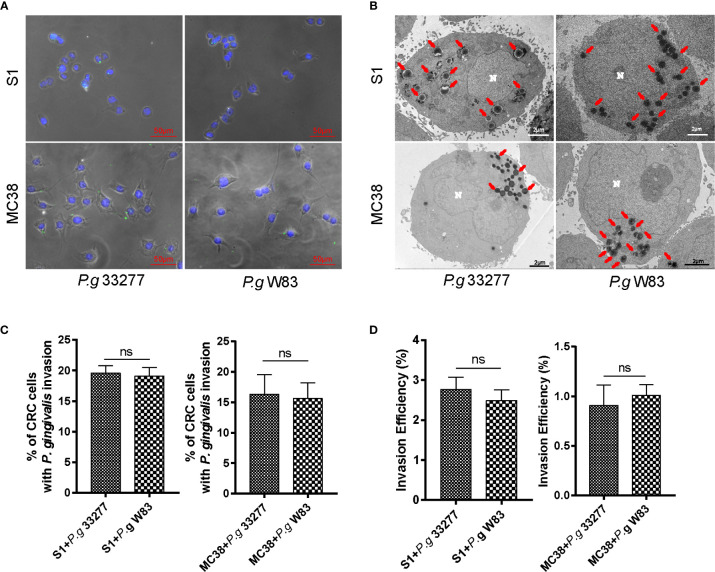
*P. gingivalis* exhibits high adhesion and invasive abilities toward colorectal cancer (CRC) cells. **(A)** Immunofluorescence micrographs show that *P. gingivalis* (green) can adhere to S1 and MC38 cells. **(B)** -Transmission electron microscopy (TEM) results indicate that *P. gingivalis* can intracellularly invade S1 and MC38 cells. **(C)** Percentage of CRC cells with *P. gingivalis* invasion were counted in 3 random fields (1000 ×, 30–50 CRC cells per fields)) by TEM and there is no significant difference between *P. gingivalis* 33277 and *P. gingivalis* W83. **(D)** Antibiotic protection assay shows that *P. gingivalis* can invade S1 and MC38 cells and survive inside the cell at 24 h after infection. The invasion efficiency of *P. gingivalis* 33277 and *P. gingivalis* W83 showed no significant difference. Invasion efficiency (%) = CFU of *P. gingivalis* inside CRC cells/CFU of *P. gingivalis* in initial inoculum. CFU, colony forming units. Red arrows, *P. gingivalis* infection in the cytoplasm. N, nucleus. ns, nonsignificant.

**Figure 2:**
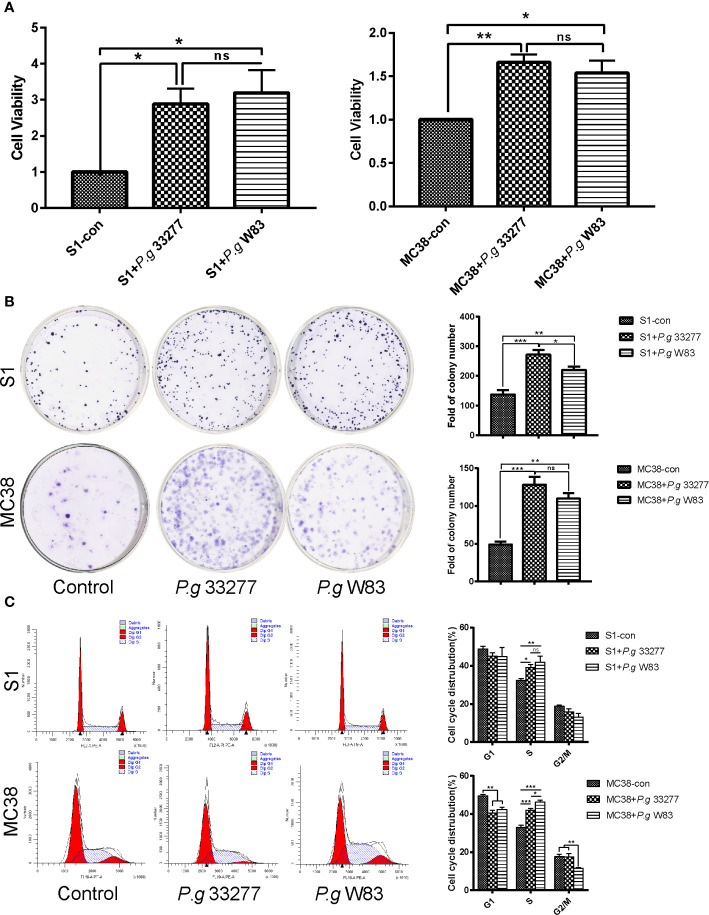
Cell proliferation and cell cycle analyses of CRC cells pretreated with *P. gingivalis*. **(A)** CCK8 assay examination of the proliferation of S1 and MC38 cells pretreated with *P. gingivalis* 33277 and W83 for 24 h. **(B)** Colony formation assay examination of the proliferation of S1 and MC38 cells pretreated with *P. gingivalis* 33277 and W83. **(C)** Cell cycle analysis of S1 and MC38 cells pretreated with *P. gingivalis* 33277 and W83 detected by flow cytometry. The results showed that the fraction of S phase cells was significantly increased. *P < 0.05, **P < 0.01, ***P < 0.001. ns, nonsignificant.

**Figure 3:**
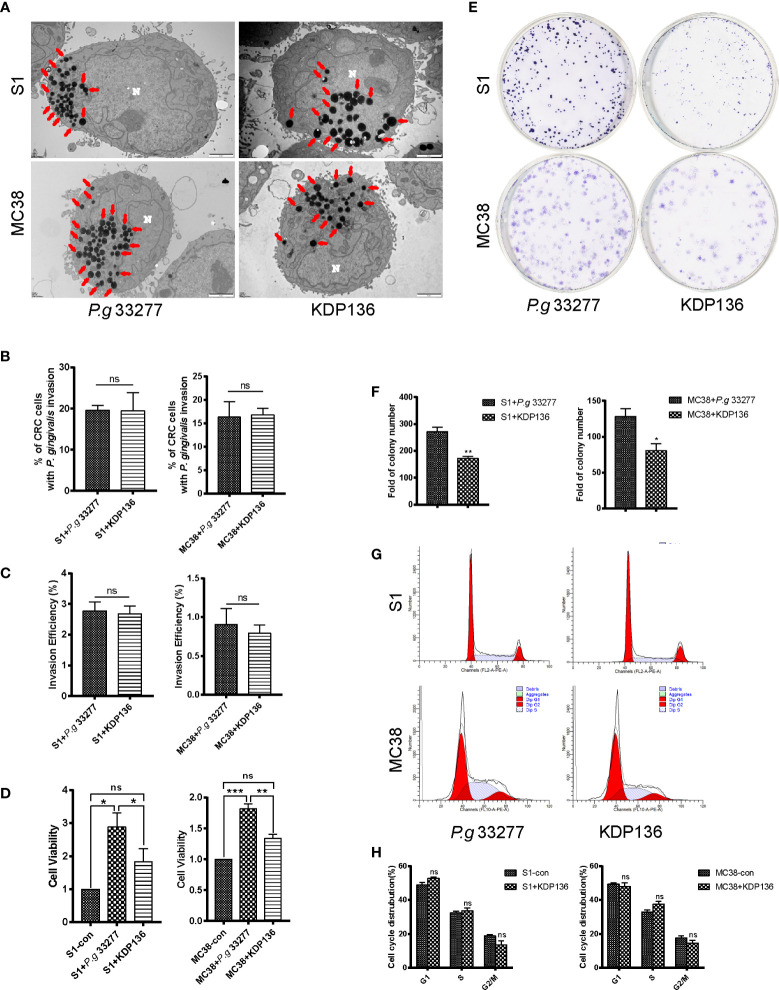
The invasion ability and pro-proliferation ability of KDP136 to S1 and MC38 cells. **(A, B)** TEM results indicate that *P. gingivalis* 33277 and KDP136 can intracellularly invade S1 and MC38 cells and the percentage of CRC cells with *P. gingivalis* invasion showed no significant difference between *P. gingivalis* 33277 and KDP136 groups. **(C)** Antibiotic protection assay shows that P. gingivalis 33277 and KDP136 can survive inside the cell at 24 h after infection and the invasion efficiency of P. gingivalis 33277 and KDP136 showed no significant difference. **(D)** CCK8 assay examination of the proliferation of S1 and MC38 cells pretreated with *P. gingivalis* 33277 and KDP136 for 24 h. **(E, F)** Colony formation assay examination of the proliferation of S1 and MC38 cells pretreated with *P. gingivalis* 33277 and KDP136. **(G, H)** Cell cycle analysis of S1 and MC38 cells pretreated with *P. gingivalis* 33277 and KDP136 detected by flow cytometry. Invasion efficiency (%) = CFU of *P. gingivalis* inside CRC cells/CFU of *P. gingivalis* in initial inoculum. CFU, colony forming units. Red arrows, *P. gingivalis* infection in the cytoplasm. N, nucleus. *P < 0.05, **P < 0.01, ***P < 0.001. ns, nonsignificant. KDP136, a gingipain-deficient mutant of *P. gingivalis* 33277.

All experiments were repeated at least three times independently and the results were presented as mean ± standard error of mean (SEM). In **Figures 1C, D** and **3B, C, F, H**, comparison between two groups was carried out with student’s t-test. In **Figures 2A, C**, **3D**, and **5A–D**, **Supplementary Figures 4B****,** **2A, B****,** **3B** and **5A, B**, comparison among three or more groups was determined using one-way ANOVA. All data analyses were performed using IBM SPSS Statistics 20 (SPSS Inc. Chicago, IL) and GraphPad Prism 6 (GraphPad Software Inc. la Jolla, CA).

## Results

### *P. gingivalis* Can Adhere to and Invade CRC Cells

To assess the adhesive and invasive capabilities of *P. gingivalis*, we established an acute infection model *in vitro*. Two CRC cell lines, MC38 and S1, were infected with *P. gingivalis* at a multiplicity of infection (MOI) of 100 for 6 h. Immunofluorescence tests of MC38 and S1 cells revealed colocalization of *P. gingivalis* marked by Alexa Fluor 488-labeled primary antibodies (**Figure 1A**).

To further verify the subcellular localization of internalized *P. gingivalis* and quantify the frequencies of invasion, we observed the changes in *P. gingivalis*-infected CRC cells by transmission electron microscopy (TEM). *P. gingivalis* appeared as electron-dense objects (0.3 to 0.5-μm in diameter) surrounded by an outer membrane in the cytoplasm of CRC cells (**Figure 1B**). The percentage of CRC cells infected with P. gingivalis 33277 and W83 showed no significant difference in CRC cells (**Figure 1C**). It is shown that around 19.3% S1 cells (S1+P. g 33277: 19.6%; S1+P. g W83: 19.1%) and 16.0% MC38 cells (MC38+P. g 33277: 16.3%; MC38+P. g W83: 15.6%) were infected with P. gingivalis after 24 h cocultivation. But the percentage will be higher in fact because ultrathin sections (100 nm) cannot present the whole picture of cells.

Antibiotic protection assay was carried to determine the number of *P. gingivalis* survive successfully inside the cell. The results showed that *P. gingivalis* 33277 and W83 can survive inside the cell after 24 h infection and the invasion efficiency of *P. gingivalis* 33277 and W83 showed no significantly difference (**Figure 1D**). There are 2.6% *P. gingivalis* survive successfully in S1 cells after 24 h cocultivation and it is 1.0% in MC38 cells. The invasion efficiency of *P. gingivalis* showed a wide variation in different CRC cell lines.

### *P. gingivalis* Promotes CRC Cell Proliferation and Increases the Percentage of CRC Cells in S Phase *In Vitro*

We performed a cell counting kit-8 (CCK8) assay and found changes in the proliferation of MC38 and S1 cells following infection with *P. gingivalis* 33277 and W83. The results indicated that *P. gingivalis* can significantly promote S1 and MC38 cell proliferation (*P* < 0.05), and no significant difference this activity was observed between the two *P. gingivalis* strains (**Figure 2A**).

Colony formation assays were performed to investigate the proliferative abilities of the CRC cell lines. Interestingly, S1 and MC38 cells co-cultured with *P. gingivalis* formed significantly more total colonies compared with that observed when CRC cells were cultured alone (*P* < 0.05), while no significant difference was observed among the experimental groups in MC38 cells (**Figure 2B**).

The S phase fraction is an important measure of cell proliferative activity. To determine whether *P. gingivalis* specifically associated with CRC cells of a specific cell cycle phase, cells were stained with propidium iodide, and the cell cycle was analyzed by flow cytometry. The ratio of S1 and MC38 cells in S phase was notably higher in the *P. gingivalis* infected groups than that observed in the uninfected control group (*P* < 0.05). No significant difference was observed between the percentage of S phase cells in the *P. gingivalis* 33277 and *P. gingivalis* W83 groups (*P* > 0.05) (**Figure 2C**). Remarkably, *P. gingivalis* 33277 and W83 could promote CRC cells proliferation and increase the percentage of CRC cells in S phase.

### *P. gingivalis* Gingipains Play an Important Role in Promoting the Proliferation of CRC Cells

To assess the effect of gingipains on CRC cell lines, we used the gingipain-deficient mutant KDP136 (Δ*rgpA*Δ*rgpB*Δ*kgp*). The invasion ability of KDP136 was assessed by TEM, and the results indicate that KDP136 can intracellularly invade S1 and MC38 cells and the percentage of CRC cells infected with *P. gingivalis* showed no significant difference between *P. gingivalis* 33277 and KDP136 groups (**Figures 3A, B**). To further quantify the invasion ability of *P. gingivalis*, we performed antibiotic protection assay. The results showed that KDP136 can survive successfully inside the S1 and MC38 cells and the invasive efficiency of KDP136 and *P. gingivalis* 33277 showed no significant difference after infection of 24 h (**Figure 3C**).

For CCK8 assay, the cell viability of the KDP136 group showed no significant differences with that observed in the control group, whereas a significant (*P*<0.05) difference was observed between the KDP136 and *P. gingivalis* 33277 groups (**Figure 3D**). To further verify the role of gingipain, we added exogenous gingipains with different concentrations of 1, 2, 3, 4 and 5 U/L. Exogenous gingipains were added at the beginning of infection. We found that the cell vitality of KDP136 group with exogenous gingipains (5 U/L) is significantly higher than control group (**Supplementary Figure 2**).

For the colony formation assay, KDP136 had a lower ability to promote S1 and MC38 cell proliferation compared with *P. gingivalis* 33277 (**Figures 3E, F**). The percentage of S phase cells in the KDP136 group was significantly lower than that observed in the *P. gingivalis* 33277 group (**Figures 3G, H**). KDP136 can invade CRC cells and survive successfully inside cytoplasm after 24 h cocultivation and the invasive ability of KDP136 are similar to *P. gingivalis* 33277. But as a gingipain-deficient mutant strain of *P. gingivalis* 33277, KDP136 has less of an ability to enhance cell viability and stimulate the cell cycle of CRC cells. Thus, gingipain plays an important role in promoting the proliferation of CRC cells.

### *P. gingivalis* Can Potentially Promote Pathways Associated With Cell Proliferation

**Figure 4:**
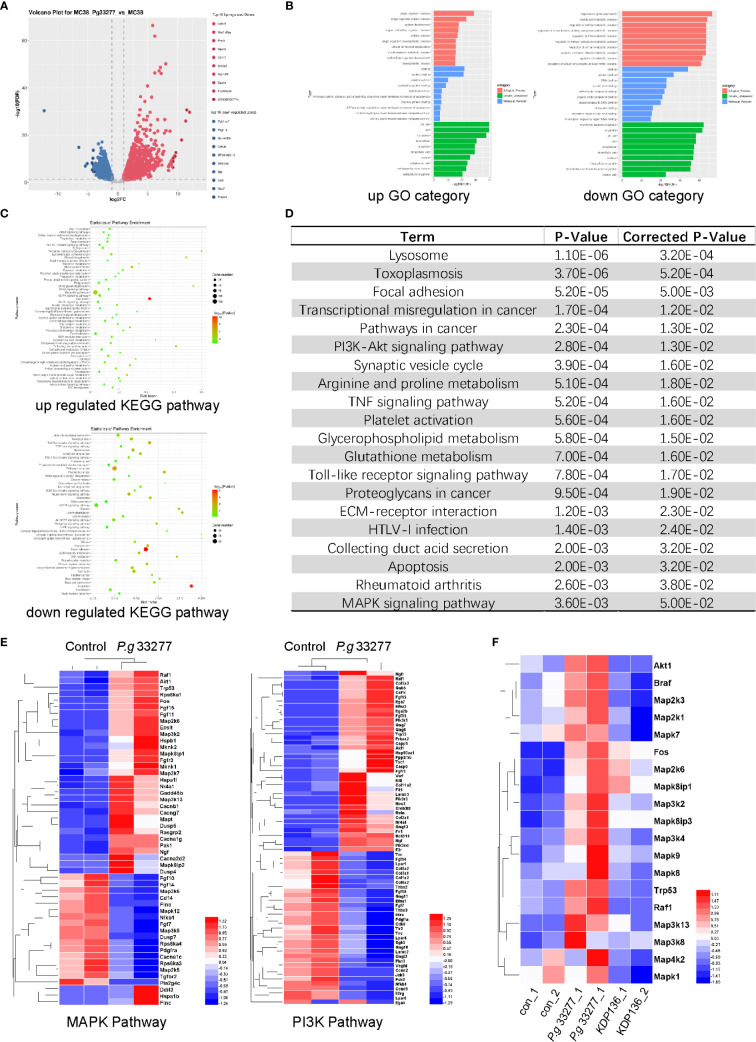
RNA-Seq results of MC38 cells pretreated with *P. gingivalis* for 24 h. **(A)** Volcano plot of gene expression differences between the *P. gingivalis* and control groups. **(B)** The distribution of differentially expressed genes (DEGs) within the Gene Ontology (GO) categories. Left, upregulated genes; right, downregulated genes. **(C)** The significantly enriched Kyoto Encyclopedia of Genes and Genomes (KEGG) pathways. Top, upregulated pathways; bottom downregulated pathways. **(D)** Genes with significant differences were analyzed with the KEGG database, and the top 20 pathways that were significantly altered are shown. **(E)** Differentially expressed genes (DEGs) between *P. gingivalis* 33277 group and Control group in the MAPK and PI3K pathways. **(F)** The expression of MAPK pathway-related genes in the MC38, *P. gingivalis* 33277 and KDP136 groups.

RNA was extracted from MC38 cells infected with *P. gingivalis* 33277 (24 h, MOI=100) for RNA-Seq analysis, the results of which were used to perform functional enrichment analysis, including Gene Ontology and Kyoto Encyclopedia of Genes and Genomes (KEGG) analyses. The results showed that there were 3292 differentially expressed genes (DEGs) between the *P. gingivalis* 33277 and control groups, of which 1893 were upregulated and 1399 were downregulated (**Figure 4A**). To determine the functions of the identified DEGs, these genes were analyzed using the Gene Ontology database, resulting in the identification of three ontologies, namely, biological process, cellular component and molecular function (**Figure 4B**). We further analyzed the significantly enriched pathways using the KEGG database. When comparing the *P. gingivalis* 33277 and control groups, 51 pathways were upregulated and 44 pathways were downregulated (**Figure 4C**), and the top 20 pathways are shown in **Figure 4D**. Included among these pathways were proliferation related pathways, including the PI3K-Akt signaling and MAPK signaling pathways (Fang and Richardson, 2005; Schmidt et al., 2020). Moreover, as shown in **Figure 4E**, we compared DEGs associated with the PI3K-Akt and MAPK signaling pathways between *P. gingivalis* 33277 and the control groups (the Log2 Fold Change” and “Adjusted P-Value” information are shown in **Supplementary Table 2**). In addition, compared to *P. gingivalis* 33277, KDP136 lost the ability to activate MAPK pathway-related genes (**Figure 4F**, the Log2 Fold Change” and “Adjusted P-Value” information are shown in **Supplementary Table 3**). Thus, we observed that the PI3K-Akt and MAPK signaling pathways are potentially involved in the interaction between *P. gingivalis* and CRC cells.

### *P. gingivalis* Promotes CRC Cell Proliferation *via* Activation of the MAPK/ERK Pathway

**Figure 5:**
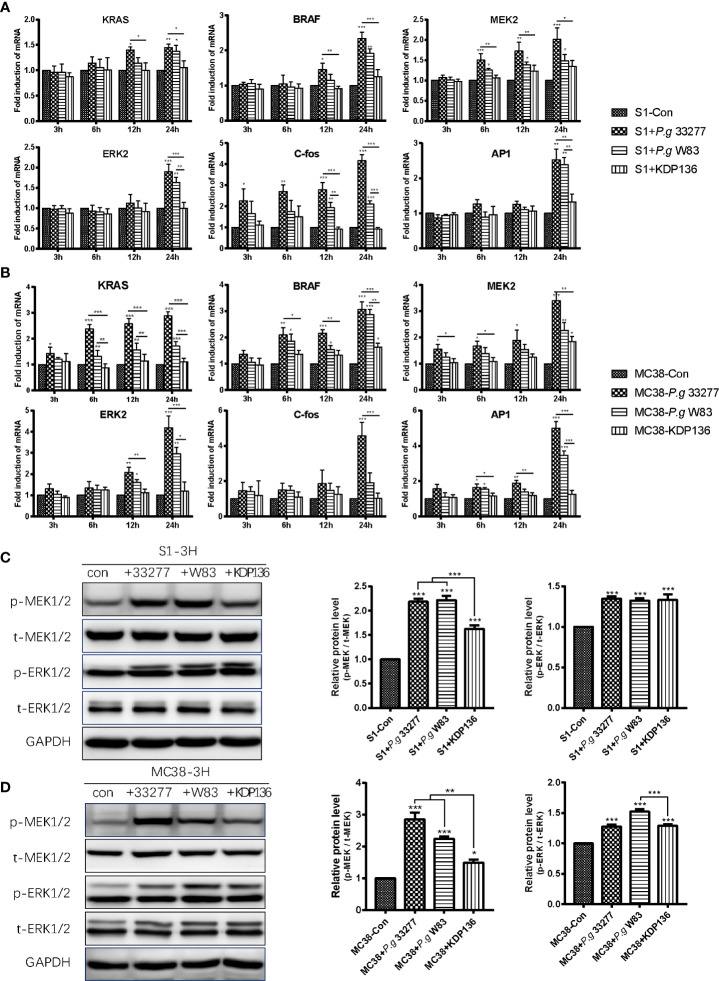
*P. gingivalis* can promote the proliferation of S1 and MC38 cells through the MAPK/ERK pathway. **(A, B)** The mRNA levels of *KRAS*, *BRAF*, *MEK2*, *ERK2*, *C-Fos* and *AP1* in S1 cells **(A)** and MC38 cells **(B)** were detected by quantitative polymerase chain reaction (qPCR) at 3, 6, 12 and 24 h after infection. **(C, D)** Western blot analysis was performed to assess the levels of the MAPK/ERK pathway-related proteins p-MEK1/2, t-MEK1/2, p-ERK1/2, t-ERK1/2 in S1 cells **(C)** and MC38 cells **(D)** at 3 h after infection. The protein levels were normalized to GAPDH. p-MEK1/2, phospho-MEK1/2. t-MEK1/2, total MEK1/2. p-ERK1/2, phospho-ERK1/2. t-MEK1/2, total ERK1/2. *P < 0.05, **P < 0.01, ***P < 0.001.

Overexpression and activation of the MAPK/ERK pathway plays an important role in the progression of CRC. Furthermore, activation of the RAS/RAF/MEK/ERK axis is crucial for the ability of the MAPK/ERK pathway to regulate various cellular responses, including the stimulation of C-fos and AP1 (Fang and Richardson, 2005). The RNA-Seq results showed that *Raf1*, *Braf*, and *Fos* levels were upregulated in the *P. gingivalis* 33277 group, whereas changes in the expression of *RAS*, *MEK*, *ERK* and *AP1* were unclear (**Figures 4E, F**). We extracted RNA from MC38 and S1 cells exposed to three *P. gingivalis* stains for quantitative polymerase chain reaction (qPCR) analysis (Gene count of all groups are shown in **Supplementary Material**). In general, our results showed that *KRAS*, *BRAF*, *MEK2*, *ERK2*, *C-fos* and *AP1* levels in the *P. gingivalis* 33277 and *P. gingivalis* W83 groups were higher than those observed in the control group after exposure to bacteria for different amounts of time. Specifically, the expression levels of *KRAS* and *BRAF*, upstream components of the MAPK/ERK pathway, were upregulated at the early stage (3 and 6 h) of the infection. *MEK2* and *ERK2* levels were significantly higher at 6 and 12 h after infection. As downstream components of the MAPK/ERK pathway, *C-fos* and activator protein-1 (*AP1*) levels were upregulated at 12 and 24 h after infection (**Figures 5A, B**). To assess the protein level and phosphorylation at the early stage of infection, we isolated protein from S1 and MC38 cells after infection with *P. gingivalis* for 3 h. The expression of total MEK1/2 (t- MEK1/2), phospho-MEK1/2 (p-MEK1/2), total ERK1/2 (t- ERK1/2), and phospho-MEK1/2 (p-ERK1/2) was measured by western blot (WB). No significant difference was observed between the experimental and control groups in t- MEK1/2 and t- ERK1/2 expression. However, regarding the phosphorylation levels of these proteins, p-MEK/t-MEK and p-ERK/t-ERK levels were significantly increased (**Figures 5C, D**). Thus, the transcription of genes encoding components in the entire MAPK/ERK pathway was gradually activated by *P. gingivalis* during the first 24 h after infection, while the phosphorylation of components in this pathway was stimulated in the first 3 h after infection.

Furthermore, the expression of PI3K-Akt pathway-related proteins in the S1 and MC38 cells was measured by WB, and the results showed that *P. gingivalis* 33277 had no effect on p-Akt (308), p-Akt (473) and Akt (pan) levels after 3, 6 and 12 h of infection (**Supplementary Figure 3**).

Unsurprisingly, KDP136 failed to stimulate the transcription of *KRAS*, *BRAF*, *MEK2*, *ERK2*, *C-fos*, *AP1* in S1 and MC38 cells (**Figures 5A, B**). In addition, the levels of p-MEK1/2 in the KDP136 group and p-ERK1/2 in the S1+KDP136 group were significantly lower than those observed in the *P. gingivalis* 33277 and W83 groups (**Figures 5C, D**). Gingipain plays an important role in regulating the MAPK/ERK pathway. Remarkably, KDP136 can significantly increase MEK1/2 and ERK1/2 phosphorylation, prompting the expression of other *P. gingivalis* virulence factors that may also influence MEK1/2 and ERK1/2 phosphorylation in addition to gingipain (**Figures 5C, D**). Previous studies showed that *P. gingivalis*-LPS, a bacterial endotoxin located on the lateral lobule of bacterial adventitia, can activate Toll like receptor 4 (TLR4) and Toll like receptor 2 (TLR2) simultaneously after recognized (Darveau et al., 2004; Nichols et al., 2009; Jia et al., 2019). As one of the adaptors of TLR2/4, MYD88 can activate MAPK pathway indirectly (Liu et al., 2014). So the LPS of KDP136 may increase the expression of p-MEK and p-ERK here.

## Discussion

*P. gingivalis* is capable of adhering to and invading host cells, and epithelial cells of the oral mucosa are considered to be the most important intracellular niche for *P. gingivalis* (Colombo et al., 2007; Lee et al., 2018). There are four phases of successful bacterial invasion: (a) entry, (b) survival, (c) replication, and (d) exit from the host cell (Casadevall, 2008). Thus, entering cells is the first step to interact with host cells for intracellular pathogens. Once invaded, this intracellular opportunistic pathogen can manipulate the host machinery to promote its long-term survival. In our results, *P. gingivalis* 33277 could tightly adhere to CRC cells after cocultivation at 4°C for 30 min. Over time, an increasing number of *P. gingivalis* cells entered the host cells, and almost all of them were located in the cytoplasm at 24 h after infection. This finding was consistent with that of a previous study on the invasion ability of *P. gingivalis* toward epithelial cells using a wide-field deconvolution microscopy technique (Belton et al., 1999).

Interestingly, the adhesion ability of *P. gingivalis* W83 and KDP136 which is detected by flow cytometry are obviously lower than *P. gingivalis* 33277 (**Supplementary Figure 4**), but there is no significant difference between *P. gingivalis* 33277 and W83 in invasion detection, promoting the proliferation and activating MAPK/ERK signaling pathway of CRC cells (**Figures 1B, C**, **Figure 2**, **Figure 5**). Firstly, the adhesion ability of *P. gingival*is is related to fimbriae, which is composed of FimA and Mfa1. *P. gingivalis* gingipain were also involved in the biosynthesis process of fimbriae (Weinberg et al., 1997). It is reported that FimA and Mfa1, major subunit proteins of long and short fimbriae, were abundant components of 33277 but not W83 (Mantri et al., 2015). This is why the adhesion ability of *P. gingivalis* W83 (less FimA and Mfa1) is similar to KDP136 (lack of gingipain). Secondly, the cultivation system of *P. gingivalis* and CRC cells *in vitro* is stable while *P. gingivalis* will be washed away by silva and intestinal content in human digestive tract. We supposed that *P. gingivalis* can invade host cells without adhesion *in vitro*, which is different with it *in vivo*. Finally, *P. gingivalis* were co-cultured with CRC cells for 24 h (37°C) in invasion assay while the adhesion assay was carried in 30 min (4°C) after infection. So the variation of adhesion ability after 30 min infection has no influence on our acute infection model (24 h co-culture) *in vitro*. It is reported that around 9–10% of osteoblasts were infected by *P. gingivalis* 33277 after cocultivation (7 d, MOI=200) detected by confocal fluorescence microscopy (Zhang et al., 2010). Another research indicated that 5.4% of gingival fibroblasts were infected by *P. gingivalis* 33277 after cocultivation (4 h, MOI=1000) detected by confocal fluorescence microscopy (Jang et al., 2017). In this study, around 19.3% S1 cells and 16.0% MC38 cells were infected with *P. gingivalis* (S1+P. g 33277: 19.6%, S1+P. g W83: 19.1%, MC38+P. g 33277: 16.3%, MC38+P. g W83: 15.6%) after cocultivation (24 h, MOI=100) which is higher than previous study (**Figures 1C**, **3B**). The percentage will be higher in fact because ultrathin sections (100 nm) cannot present the whole picture of cells. We supposed that CRC cells are one of the desirable niches for *P. gingivalis*.

*P. gingivalis* has been reported to activate the p38MAPK/HSP27, ERK1/2-Ets1, and PAR2/NF-kB pathways to stimulate the expression of promatrix metalloproteinase-9 (proMMP-9), and its ability to invade OSCC cell lines was promoted by gingipain (Inaba et al., 2014). Furthermore, *P. gingivalis* was shown to improve the proliferation ability and upregulated the percentage of S phase cells in human immortalized oral epithelial cells using a long-term infection model (MOI=1, 5–23 weeks) (Geng et al., 2017). Considering that long-term exposure to aerobic environment will dramatically reduce the viability of *P. gingivalis*, we established an acute infection model (MOI=100, <24 h) to ensure that the majority of cells for this obligate anaerobe were alive.

Proteomic analysis showed that *P. gingivalis* elevated the level of Cyclin A to promote the proliferation of gingival epithelial cells (Kuboniwa et al., 2008). Increased expression of cyclin D1 were also detected in OSCC cells infected with *P. gingivalis*. Cyclin D1 contribute to the enhanced proliferation of OSCC cells, which was recognized as an early event of oral carcinogenesis (Ramos-García et al., 2019). Another research showed that *P. gingivalis* promotes the G1/S transition from 6 h to 12 h after infection by up-regulating the expression of cyclin D and cyclin E (Pan et al., 2014). Overall, *P. gingivalis* can regulates cell cycle to enhance the proliferation of OSCC cells and gingival epithelial cells (Kuboniwa et al., 2008). In our model, *P. gingivalis* could also promote the proliferation and improve the percentage of CRC cells in S phase at 24 h after infection. The expression of AP1, a dimer combined with proteins from Fos and Jun sub-families, is up-regulated by *P. gingivalis* in our study (**Figures 5A, B**). As an important transcription factor, AP1 can influence the expression of cyclin D1 (Vartanian et al., 2011) and regulate the cell cycle of CRC cells.

Gingipain is a potent *P. gingivalis* virulence factor that targets several essential components in the human immune system, coagulation cascade, and regulatory pathways (Imamura et al., 2003). *P. gingivalis* can activate the ERK1/2-Ets1, p38/HSP27, and PAR2/NFκB pathways to induce promatrix metalloproteinase-9 expression after invading OSCC cells. Then, proMMP-9 is released into the extracellular environment by gingipain from *P. gingivalis via* PAR2 activation to promote cellular invasion and metastasis (Inaba et al., 2014). MMP-9 is known as a type IV collagenase that is associated with various physiological and pathological processes, including reproduction, growth, development, inflammation, and vascular and proliferative diseases (Van den Steen et al., 2002). MMP-9 transcription is positively regulated by E-26 transcription factors, NFκB, polyomavirus enhancer A-binding protein-3, and AP1 (Crawford and Matrisian, 1996). AP1 was upregulated by gingipain in our acute model in the present study (**Figures 5A, B**). Thus, gingipain may activate proMMP-9 by improving AP1 expression to promote CRC progression.

Increasing evidence has revealed that the MAPK/ERK signaling pathway plays a key role in CRC cell proliferation, migration, invasion, apoptosis and differentiation (Sun et al., 2019; Cheng et al., 2019; Pan et al., 2019). It has been reported that RAF, including its three isoforms (ARAF, BRAF, and CRAF), is activated by RAS and then activates MEK1/2 by increasing its phosphorylation level. Activated MEK1/2 (p-MEK1/2) can increase ERK1/2 phosphorylation. Finally, p-ERK1/2 can increase the expression of AP1, a transcription factor that consists of FOS and JUN, to regulate cell processes (Kim and Choi, 2015; Baxter et al., 2017). According to our RNA-Seq, qPCR and WB results, the RAS/RAF/MEK/ERK signaling pathway was stimulated by *P. gingivalis* through gingipain within 24 h. In addition, the phosphorylation of MEK1/2 and ERK1/2, the core pathway components, was upregulated within 3–6 h. Thus, we can conclude that *P. gingivalis* probably promotes the proliferation of CRC cells by regulating the MAPK/ERK signaling pathway.

It is reported that *F. nucleatum are* always work together with *P. gingivalis* in mouse experimental periodontitis model (Polak et al., 2009). We compare the pro-proliferation ability of *Fusobacterium nucleatum* (*F. nucleatum*) and *P. gingivalis* using CCK8 assay. The results showed there is no significant synergism or summation action between *F. nucleatum* and *P. gingivalis* in our acute infection model *in vitro* (**Supplementary Figure 5**). So we haven’t focus on the interaction between *F. nucleatum* and *P. gingivalis* in our study. It is our next step to explore the synergism or summation action between *F. nucleatum* and *P. gingivalis*.

Overall, this study provides a direct evidence for the association of *P. gingivalis* and CRC cells *in vitro*. Animal experiment and retrospective analysis of clinical cases are required to confirm our conclusion. It suggests that *P. gingivalis* can potentially promote CRC progression and CRC patients with *P. gingivalis* infection requires extra attention in clinical work.

## Data Availability Statement

All datasets generated for this study are included in the article/**Supplementary Material**.

## Author Contributions

ZW and BC contributed to conception and design of the experiment and critically revised the manuscript. WM and YJ performed the experiments and analyzed the data. XC and HL drafted the manuscript. All authors contributed to the article and approved the submitted version.

## Funding

This project was supported by grants from National Natural Science Foundations of China (No. 81630025 and 81700979) and Special Funds for the Cultivation of Guangdong College Students’ Scientific and Technological Innovation (“Climbing Program” Special Funds, pdjh2020a0004).

## Conflict of Interest

The authors declare that the research was conducted in the absence of any commercial or financial relationships that could be construed as a potential conflict of interest.

## Abbreviations

F. nucleatum, Fusobacterium nucleatum; OSCC, oral squamous cell carcinoma; CRC, colorectal cancer; *P. gingivalis*, *Porphyromonas gingivalis*; Rgp, arginine-specific protease; Kgp, lysine-specific protease; CFU, colony forming units; MOI, multiplicity of infection; FITC, fluorescein isothiocyanate; TEM, transmission electron microscope; PI, propidium iodide; CCK8, cell counting kit-8; AP1, activator protein-1; U/L, unit/L; KEGG, Kyoto Encyclopedia of Genes and Genomes; DEG, differentially expressed genes; WB, western blot; qPCR, quantitative polymerase chain reaction; proMMP-9, promatrix metalloproteinase-9; PAR2, proteinase-activated receptor 2.
