# Supplementary material for: Intracellular Porphyromonas gingivalis Promotes the Proliferation of Colorectal Cancer Cells via the MAPK/ERK Signaling Pathway
Source: Front Cell Infect Microbiol. 2020 Dec 23;10:584798. doi: 10.3389/fcimb.2020.584798 (PMC7785964; doi:10.3389/fcimb.2020.584798)
Supplement: Supplementary file 1 [file DataSheet_1.pdf]

# Supplementary Figure 1

A.

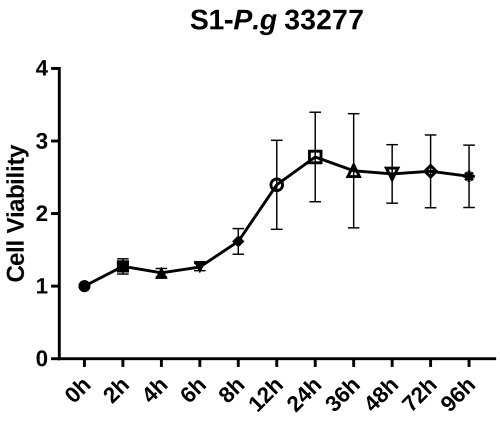


B.

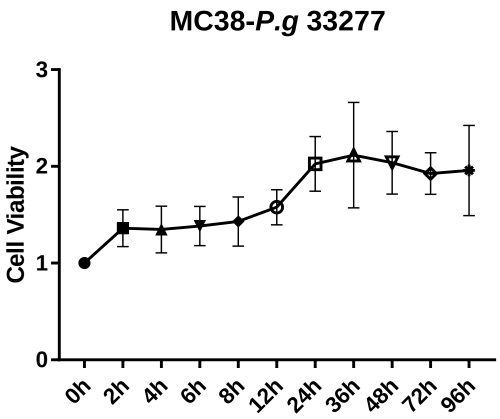


**Supplementary Figure 1.** Cell proliferation of colorectal cancer cells pretreated with *P. gingivalis* 33277 for 0-96 h. **(A-B)** Cell counting kit-8 assay shows that *P. gingivalis* can promote the proliferation of CRC cells in a time dependent manner in 0-24 h, and the cell viability of CRC cells reached a plateau in 24-96 h incubation.
