# Supplementary material for: Intracellular Porphyromonas gingivalis Promotes the Proliferation of Colorectal Cancer Cells via the MAPK/ERK Signaling Pathway
Source: Front Cell Infect Microbiol. 2020 Dec 23;10:584798. doi: 10.3389/fcimb.2020.584798 (PMC7785964; doi:10.3389/fcimb.2020.584798)
Supplement: Supplementary file 2 [file DataSheet_2.pdf]

Supplementary Figure 3.

A.

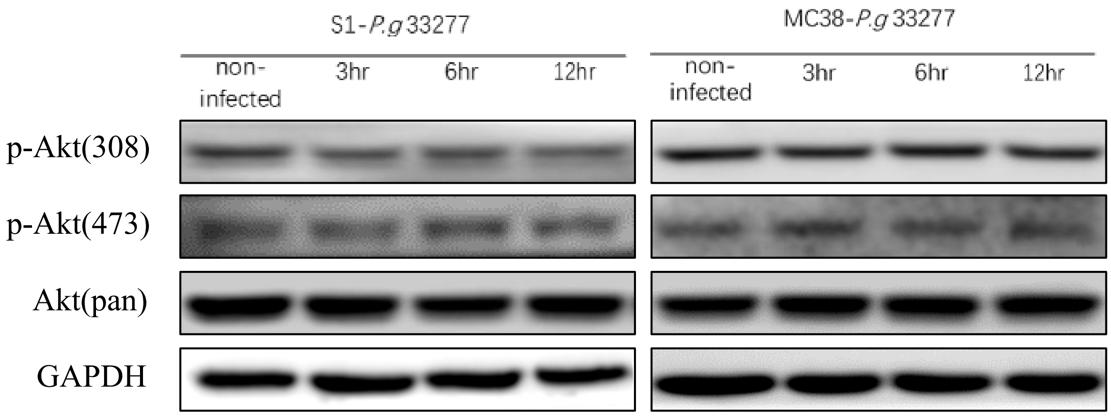

B.

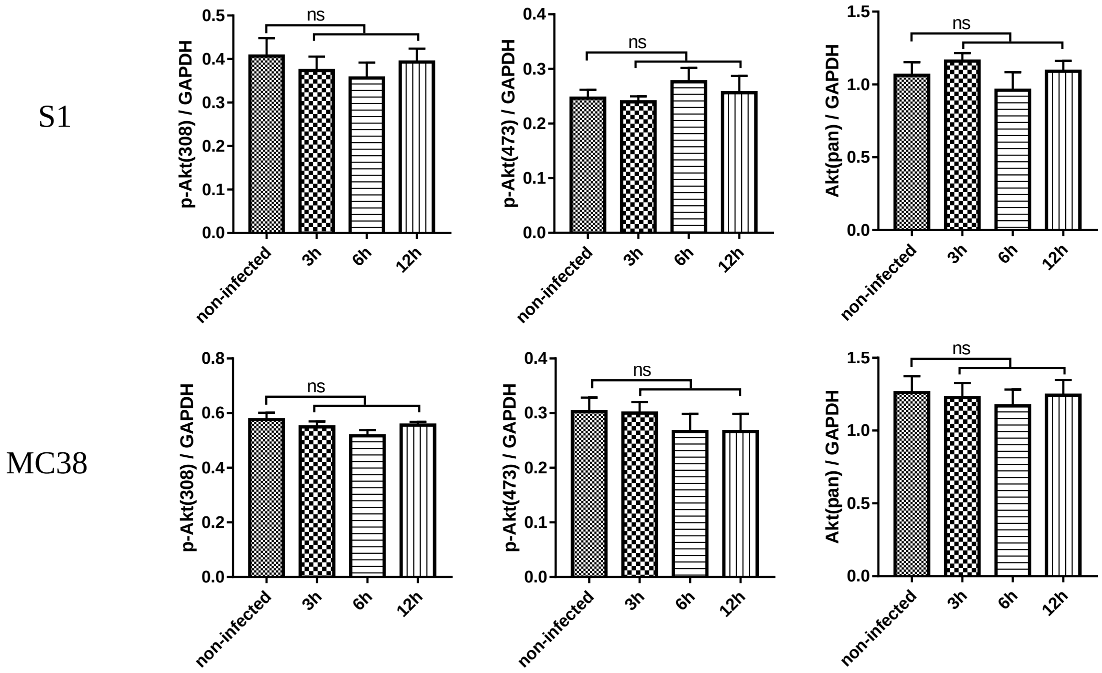

Supplementary Figure 3. The expression of PI3K-AKT pathway related protein. **(A-B)** Western blot was performed to detect the expression of p-Akt (308), p-Akt (473) and Akt (pan) in S1 cells and MC38 cells at 3h, 6h, and 12h after infection. ns, non-significant.
