# Supplementary material for: Intracellular Porphyromonas gingivalis Promotes the Proliferation of Colorectal Cancer Cells via the MAPK/ERK Signaling Pathway
Source: Front Cell Infect Microbiol. 2020 Dec 23;10:584798. doi: 10.3389/fcimb.2020.584798 (PMC7785964; doi:10.3389/fcimb.2020.584798)
Supplement: Supplementary file 3 [file DataSheet_3.pdf]

Supplementary Figure 4

A.

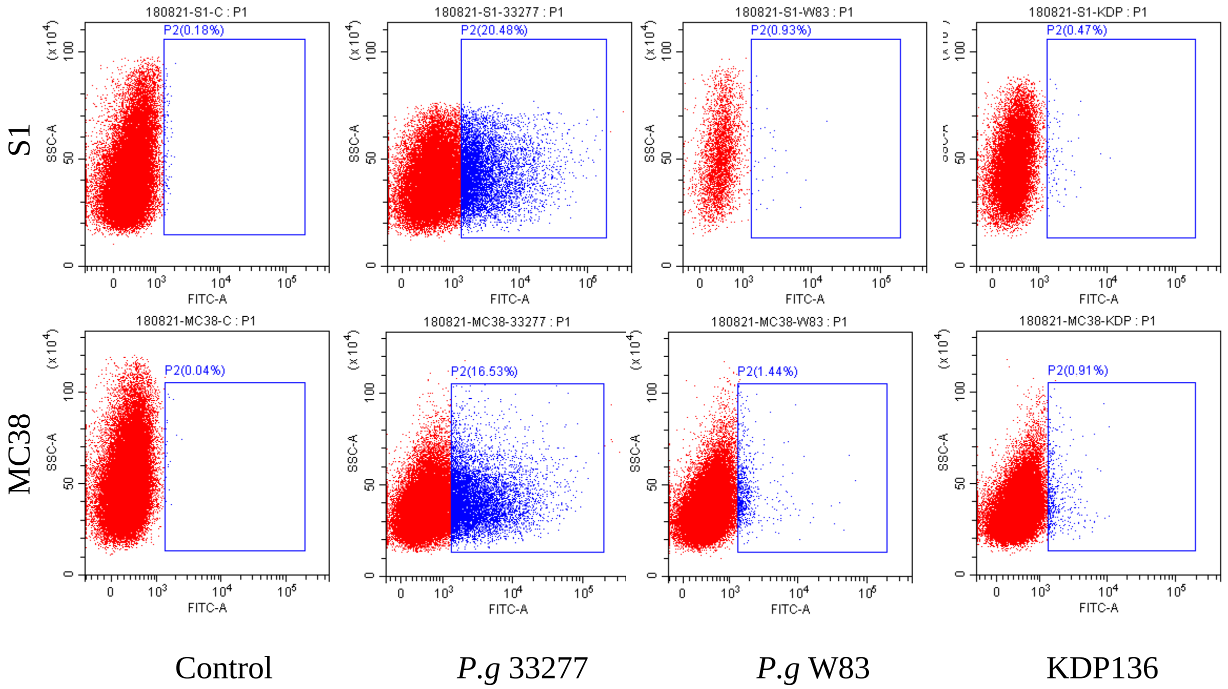

B.

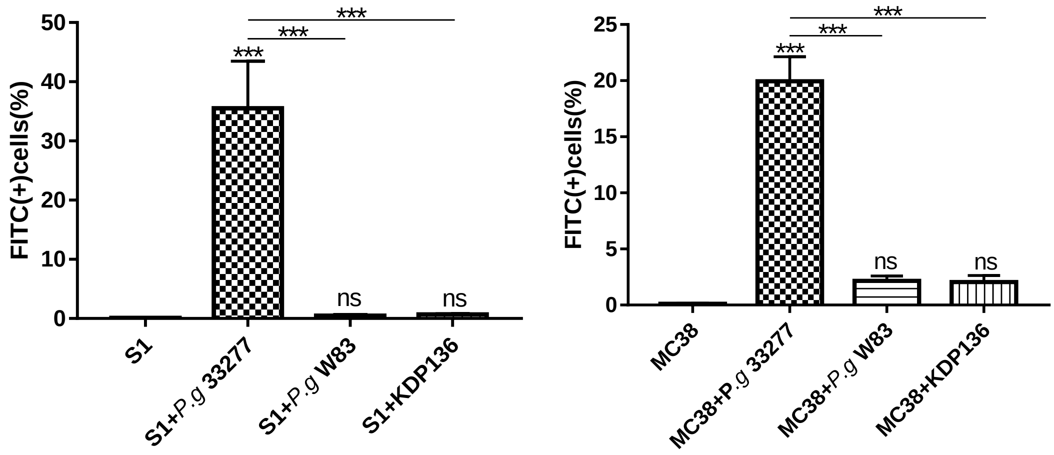

Supplementary Figure 4. Adhesive ability of *P. gingivalis* 33277, *P. gingivalis* W83 and KDP136 to colorectal cancer (CRC) cells. **(A-B)** Flow cytometry results indicate *P. gingivalis* 33277 have significantly (P<0.001) higher adhesion ability than *P. gingivalis* W83 and KDP136. ***P<0.001. ns, non-significant.
