# Supplementary material for: Intracellular Porphyromonas gingivalis Promotes the Proliferation of Colorectal Cancer Cells via the MAPK/ERK Signaling Pathway
Source: Front Cell Infect Microbiol. 2020 Dec 23;10:584798. doi: 10.3389/fcimb.2020.584798 (PMC7785964; doi:10.3389/fcimb.2020.584798)
Supplement: Supplementary file 4 [file DataSheet_4.pdf]

Supplementary Figure 5.

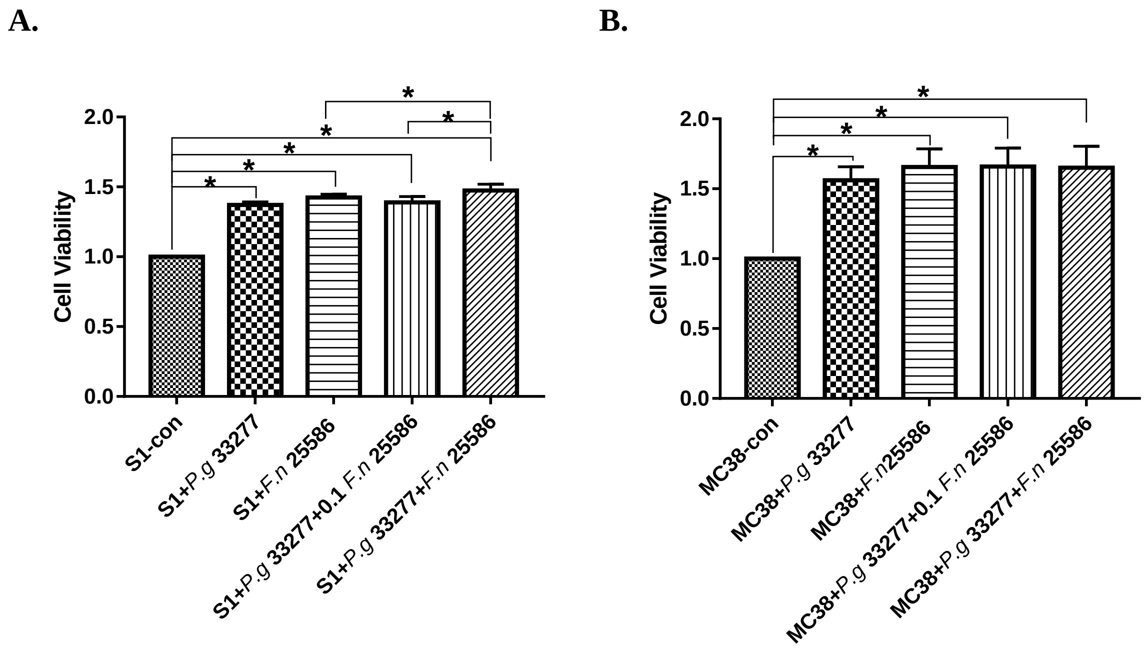


**Supplementary Figure 5.** Cell counting kit-8 (CCK8) results of S1 and MC38 pretreated with *P. gingivalis* and *F. nucleatum* for 24 h. CRC cells were incubated with *P. gingivalis 33277* and *F. nucleatum 25586* at a MOI of 100. Expectedly, the MOI in “0.1 *F. nucleatum 25586*” group is 10. *F.nucleatum* 25586 was purchased from ATCC. *$P<0.05$.
