# Supplementary material for: Intracellular Porphyromonas gingivalis Promotes the Proliferation of Colorectal Cancer Cells via the MAPK/ERK Signaling Pathway
Source: Front Cell Infect Microbiol. 2020 Dec 23;10:584798. doi: 10.3389/fcimb.2020.584798 (PMC7785964; doi:10.3389/fcimb.2020.584798)
Supplement: Supplementary file 5 [file DataSheet_5.pdf]

Supplementary Table 1. Sequence of primers used for human and mouse genes

Gene	Specie	Forward primer	Reverse primer
KRAS	Human	GGACTGGGGAGGGCTTTCT	GCCTGTTTTGTGTCTACTGTTCT
	Mouse	CAAGAGCGCCTTGACGATACA	CAAGAGCGCCTTGACGATACA
BRAF	Human	AATACACCAGCAAGCTAGATGC	AATCAGTTCCGTTCCCCAGAG
	Mouse	ATTTGGTGGAGAGCATAACCCA	ATTTGGTGGAGAGCATAACCCA
MEK2	Human	CCAAGGTCGGCGAACTCAAA	TCTCAAGGTGGATCAGCTTCC
	Mouse	GTTACCGGCACTCACTATCAAC	GTTACCGGCACTCACTATCAAC
ERK2	Human	TACACCAACCTCTCGTACATCG	CATGTCTGAAGCGCAGTAAGATT
	Mouse	GGTTGTTCCCAAATGCTGACT	GGTTGTTCCCAAATGCTGACT
C-FOS	Human	CCGGGGATAGCCTCTCTTACT	CCAGGTCCGTGCAGAAGTC
	Mouse	CGGGTTTCAACGCCGACTA	TGGCACTAGAGACGGACAGAT
AP1	Human	TCCAAGTGCCGAAAAAGGAAG	CGAGTTCTGAGCTTTCAAGGT
	Mouse	TTCCTCCAGTCCGAGAGCG	TGAGAAGGTCCGAGTTCTTGG
GAPDH	Human	GGAGCGAGATCCCTCCAAAAT	GGCTGTTGTCATACTTCTCATGG
	Mouse	AGGTCGGTGTGAACGGATTTG	AGGTCGGTGTGAACGGATTTG
