# Supplementary material for: Intracellular Porphyromonas gingivalis Promotes the Proliferation of Colorectal Cancer Cells via the MAPK/ERK Signaling Pathway
Source: Front Cell Infect Microbiol. 2020 Dec 23;10:584798. doi: 10.3389/fcimb.2020.584798 (PMC7785964; doi:10.3389/fcimb.2020.584798)
Supplement: Supplementary file 6 [file DataSheet_6.pdf]

Supplementary Table 2. The comparisons of "*P.g* 33277 vs Control"

	Log2 Fold Change	Adjusted P-Value
Ngfr	1.90139428	3.7353E-08
Raf1	1.10136331	0.00029416
Akt1	1.1236349	0.00038732
Vwf	3.05744147	7.367E-07
Col5a3	2.94060822	4.9223E-19
Tnr	-1.7020548	8.7021E-05
Kitl	1.68941684	1.3378E-05
Col6a2	-1.0240225	0.0023147
Pik3r5	4.20624426	1.1531E-12
Hsp90aa1	1.03061833	0.0118705
Fgf10	-2.620176	0.00022229
Col2a1	2.45936154	0.0031342
Nr4a1	1.99162333	3.4294E-10
Thbs2	-1.8587402	0.00135865
Col11a2	1.32054162	0.01452933
Csf1r	3.22920767	3.5519E-07
Ppp2r5b	1.41514339	6.8407E-05
Itga7	4.17447365	2.8513E-23
Fgf14	-2.8696931	0.00080632
Gng13	3.35398134	5.5153E-05
Sgk3	-1.3882798	0.02524305
Col5a2	-1.5236959	1.4495E-06
Col3a1	-2.9594728	1.0156E-08
Fn1	1.3768902	8.7437E-05
Lamc2	-1.480671	0.00440458
Lamb3	1.37508436	4.988E-05
Pkn3	-1.7710124	5.646E-08
Tsc1	1.21917677	0.00058335
Fgf7	-2.3273332	0.00018722
Bcl2l11	1.22613844	4.0456E-05
Ngf	1.6620376	1.6255E-06
Il6ra	-2.1336906	0.0007521
Efna1	-1.0124997	0.00514221
Tlr2	-1.2865923	0.02053159
Efna3	2.52503454	9.8367E-13
Thbs3	-1.0101999	0.00186473

	Log2 Fold Change	Adjusted P-Value
Nfkb1	-2.2101207	8.2987E-08
Ccne2	-1.8905142	5.2003E-06
Tnc	-1.3328594	0.01228536
Prkaa2	1.04738187	0.00677724
Pik3r3	1.75112112	6.4547E-05
Casp9	1.1042366	0.00217126
Nos3	2.31247366	0.00010564
Pdgfra	-1.4538556	0.0003755
Flt1	1.23096156	0.01716726
Col1a2	-1.140584	0.00061855
Fgf15	5.14032729	1.2622E-09
Il2rg	-1.5040697	4.1098E-05
Vegfd	-1.8656713	0.00014213
Jak3	-1.5618269	4.5493E-05
Gnb5	2.38053141	2.8799E-13
Gng11	-2.4266404	0.00027777
Lpar6	-2.3076745	0.00053482
Itga2b	1.15742372	0.010867
Creb3l3	5.46419237	2.5745E-10
Gng10	-1.0457141	0.00909558
Lpar1	-2.1978549	2.1738E-06
Gngt2	-1.0765964	0.0390713
Pik3cd	2.01731379	7.7542E-07
Cdk6	-1.4673059	0.00029723
Pck2	-1.7144012	6.4362E-09
Reln	3.68655241	0.00539093
Fgf11	2.21439195	9.6257E-08
Gng7	2.86401275	1.0985E-12
F2r	1.19862592	4.5578E-05
Lpar4	-1.3051933	0.04134123
Fgfr3	3.06394325	2.8457E-07
Cspp1	0.69358461	0.0468838
Trp53	2.23252851	9.4218E-12
Gng8	1.77877967	0.00078831
Ccnd1	-3.6447299	1.9887E-08
