# Supplementary material for: Intracellular Porphyromonas gingivalis Promotes the Proliferation of Colorectal Cancer Cells via the MAPK/ERK Signaling Pathway
Source: Front Cell Infect Microbiol. 2020 Dec 23;10:584798. doi: 10.3389/fcimb.2020.584798 (PMC7785964; doi:10.3389/fcimb.2020.584798)
Supplement: Supplementary file 7 [file DataSheet_7.pdf]

Supplementary Table 3. The comparisons of "KDP136 vs *P.g* 33277"

	**Log2 Fold Change**	**Adjusted P-Value**
**Akt1**	1.0932165	1.5075E-16
**Braf**	-0.0785576	0.91056532
**Fos**	0.16372824	0.86337824
**Map2k1**	0.00147582	0.99818093
**Map2k3**	-0.1035612	0.87795605
**Map2k6**	0.32874768	0.64105081
**Map3k13**	-0.8034716	0.36837429
**Map3k2**	-0.008773	0.99078603
**Map3k4**	-0.122308	0.85471833
**Map3k8**	0.0709853	0.94068406
**Map4k2**	0.15516613	0.83354955
**Mapk1**	0.03323438	0.96725652
**Mapk7**	-0.0826144	0.90035865
**Mapk8**	-0.0043229	0.99614534
**Mapk8ip1**	0.48487065	0.4376388
**Mapk8ip3**	0.28339984	0.68047222
**Mapk9**	0.08735837	0.90479934
**Trp53**	2.3158481	1.1678E-66
**Raf1**	1.038101	6.6648E-19
