# Supplementary material for: Intracellular Porphyromonas gingivalis Promotes the Proliferation of Colorectal Cancer Cells via the MAPK/ERK Signaling Pathway
Source: Front Cell Infect Microbiol. 2020 Dec 23;10:584798. doi: 10.3389/fcimb.2020.584798 (PMC7785964; doi:10.3389/fcimb.2020.584798)
Supplement: Supplementary file 8 [file DataSheet_8.pdf]

Supplementary Figure 2.

A.

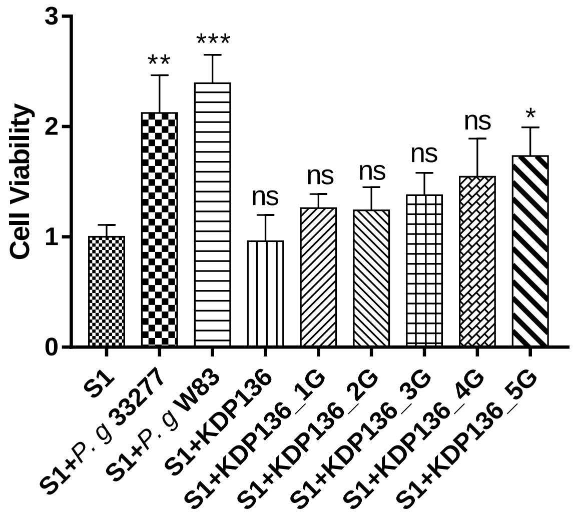

B.

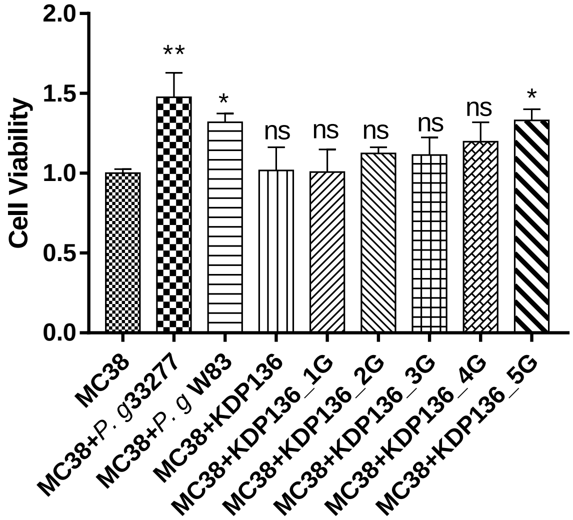

Supplementary Figure 2. Cell proliferation of colorectal cancer cells pretreated with *P. gingivalis* 33277, *P. gingivalis* W83, KDP136 and exogenous gingipains. **(A-B)** Cell counting kit-8 assay indicates that *P. gingivalis* 33277 and W83 can promote the proliferation of CRC cells and KDP136 lose the ability. However, the cell vitality of KDP136 group with exogenous gingipains (5 U/L) is significantly higher than control group. Cells were co-cultured with *P. gingivalis* at a MOI of 100 for 24 h and the exogenous gingipains were added at 0 h with different concentrations of 1, 2, 3, 4 and 5 U/L. U/L, unit/L. **P*<0.05, ***P*<0.01. ns, nonsignificant.
